# Correlation analysis of balance function with plantar pressure distribution and gait parameters in patients with cerebral infarction in the basal ganglia region

**DOI:** 10.3389/fnins.2023.1099843

**Published:** 2023-02-24

**Authors:** Sihao Liu, Huixian Yu, Zhaoxia Wang, Pei Dai

**Affiliations:** Department of Rehabilitation Medicine, Beijing Tiantan Hospital, Capital Medical University, Beijing, China

**Keywords:** stroke, walking function, balance function, plantar pressure, gait feature

## Abstract

**Objective:**

To analyze the correlation between balance function and gait parameters of patients with basal ganglia infarction. And to observe the influence of balance function on plantar pressure and hemiplegia gait based on the Berg Balance Scale (BBS) score.

**Methods:**

One hundred and forty patients with cerebral infarction hemiplegia in the basal ganglia region (a study group, *n* = 140) and healthy people (a control group, *n* = 140) were enrolled. The study group was evaluated with the BBS, the 10 m walking test (10MWT), and the timed up-and-go test (TUGT). The gait parameters and the peak plantar pressure were measured in both groups while walking, and the differences between the groups were compared. In addition, the characteristics of the plantar pressure curve of the hemiplegic and non-hemiplegic sides during walking and the correlation between the 10MWT, the TUGT, the plantar pressure peak, the gait parameters,and the BBS score were analyzed in the study group.

**Results:**

The peak plantar pressure of the forefoot and heel, stride length, lateral symmetry, stand phase, swing phase, and dual stand phase of both sides in the study group were significantly lower than those in the control group (*P* < 0.05). The BBS score negatively correlated with the 10MWT, the TUGT, the peak plantar pressure of the hemiplegic forefoot, midfoot, and the non-hemiplegic midfoot, the anterior to posterior position (ant/post position), hemiplegic stand phase, and the dual stand phase (*P* < 0.05). The BBS score positively correlated with the hemiplegic swing phase and stride length (*P* < 0.05).

**Conclusion:**

A correlation was found between the forefoot plantar pressure and the stand phase of the hemiplegic limbs, the ant/post position, and the balance function after basal ganglion cerebral infarction. This association can be used in walking and balance assessment for stroke rehabilitation. Correcting forefoot pressure or the front and ant/post position can improve balance function.

## 1. Introduction

Motor dysfunction often occurs after a stroke (Wang, 2022). About 70% of stroke-affected patients have a walking dysfunction that limits their daily life. Restoring walking ability is one of the main demands of stroke-affected patients and their families, and it is the most important and most commonly shared rehabilitation goal (Mehrholz et al., 2018).

Balance dysfunction is one of the main factors influencing walking in stroke-affected patients (Rahimzadeh Khiabani et al., 2017). Due to the decline in sensory, exercise, or information-processing ability, and muscle spasticity, decreased muscle strength, and excessive energy consumption, patients experience balance dysfunction and have a high risk of falling after a stroke (Li, 2022). Reportedly, 65% of stroke-affected patients have a history of falling, which causes muscle tissue damage and ankle sprains and affects their recovery process (Lee and Lee, 2019).

Abnormal gait in stroke-affected patients, including walking parameters, transfer ability, and plantar pressure, differs from healthy people and affects the recovery process (You et al., 2016). However, clinically evaluating the balance function after stroke is limited to dynamic and static assessment, and the balance function is an indicator of patients’ ability to walk independently (Lee et al., 2013). Most scholars characterize walking as a rhythmic movement, and few studies have focused on the effect of balance function on the dynamic plantar pressure and gait characteristics of hemiplegia after stroke (Lee et al., 2020). Lewek et al. (2014) studied the relationship between gait symmetry and balance function in stroke-affected patients but only measured the correlation between the walking weight distribution and stance time asymmetry and did not further evaluate the gravity distribution and symmetry of patients when walking. Obembe et al. (2014) suggested that balance function during walking is associated with gait speed and cadence in stroke-affected patients but did not explore the effect of balance function on plantar pressure distribution during walking.

The balance characteristics of infarcts in different brain regions are not identical, and failure to distinguish between stroke sites may affect the results of the study. Cerebral infarction in the basal ganglia area is common, and hemiplegia has a prominent gait (Alexander et al., 2009). In the present study, evaluating the balance function of patients with cerebral infarction in the basal ganglia area reduced the disturbance of balance function from other intracranial injuries. Gait analysis and plantar pressure analysis were used to evaluate the patients’ walking function. Based on previous research, the correlation between balance function and plantar pressure during walking was explored, and the effect of balance function after stroke in the basal ganglion area on the characteristics of hemiplegic gait was further examined.

## 2. Materials and methods

### 2.1. Patients

One hundred and forty patients with cerebral infarction treated in the Department of Rehabilitation Medicine of Beijing Tiantan Hospital between January 2021 and August 2022 were enrolled as a study group. Another one hundred and forty healthy people were collected as a control group. There were no significant differences in age, gender, height, or weight (Table 1). This study was approved by the ethics committee of Beijing Tiantan Hospital, Capital Medical University (KY2021-040-02).

**TABLE 1:** General situation of both groups.

Group	Sex (*n*) male/female	Age (years)	Height (cm)	Weight (kg)
Control group	96/44	58.5 ± 9.3	168.8 ± 7.5	65.2 ± 8.7
Study group	82/58	57.6 ± 10.2	168.5 ± 8.1	66.8 ± 8.6
*P*	0.082	0.463	0.691	0.116

The inclusion criteria were: (1) age 40–70 years old, (2) primary basal ganglia area cerebral infarction diagnosed by magnetic resonance imaging or computed tomography, (3) the onset of the disease was >1 month ago, (4) no sensory impairments, (5) no serious cognitive dysfunction (Mini-Mental State Examination score >26), (6) could walk 10 m or more independently, and (7) provided a signed informed consent form.

The exclusion criteria were: (1) cerebrovascular disease progression and unstable vital signs; (2) other neurological or mental diseases, such as stroke, brain trauma, or Parkinson’s disease; (3) severe heart, lung, liver, or kidney dysfunction; (4) sensory aphasia, cognitive impairment, or unable to cooperate with the evaluation and examination; (5) fractures and arthritis affecting the walking function of patients; or (6) proprioception disorders.

The suspension criteria were: (1) severe adverse reactions or inability to continue, (2) deterioration of the condition or serious complications, (3) failure to cooperate and to receive required treatment, or (4) patients and their families requesting withdrawal from the study.

### 2.2. Measurement

#### 2.2.1. The 10-m walking test

The 10-m walking test (10MWT) measures the walking ability of stroke-affected patients (Yeung et al., 2018). Patients were asked to walk 16 m forward at their fastest speed in a state of natural relaxation, after which the time they spent walking between the 3-m and the 13-m points was recorded.

#### 2.2.2. The timed up-and-go test

The timed up-and-go test (TUGT) measures metastatic ability and postural control in stroke-affected patients (Dong et al., 2021). The patients were seated in a chair, lean against the chair back, and put their hands on the armrests, after which the researcher recorded with the stopwatch from the moment the patient got up, walked for 3 m, turned around a cone, and returned to the chair. When the patient sat back on the chair, the researcher stopped recording.

#### 2.2.3. Berg Balance Scale

The Berg Balance Scale (BBS) measures the balance ability of stroke-affected patients (Blum and Korner-Bitensky, 2008). It has 14 actions, each recorded with 0–4 points according to the degree of completion, with a total score of 56 points. The higher the score, the better the balance function. Patients were guided to complete 14 actions, such as independent sitting, from sitting to standing, independent standing, and from standing to sitting.

#### 2.2.4. Dynamic plantar pressure assessment and gait analysis

The Zebris plantar pressure measurement system (Zebris FDM 1.12) was used to complete the evaluation. The participants had to take off their shoes, stand on the running platform, and hold their hands on the railings on both sides of the runway while a safety device was clamped on their chest. The runway was open, and the participant was instructed to follow it. The speed of the running platform gradually increased, and when it reached the speed at which the participant was comfortable, the participant was asked to release the railings, turn on the evaluation device, and then stop the device after walking for 30 s. The device mainly recorded the data of both lower limbs while walking, including the peak plantar pressure (N/cm^2^) of the forefoot, midfoot, and heel, and the gait parameters. The gait parameters included the proportions of the stand, swing, and dual stand phases (%), and the step width (cm), stride length (cm), and the center of plantar (COP) included the anterior to posterior (ant/post) position (mm) and lateral symmetry (mm).

### 2.3. Statistical analysis

Graph Pad Prism 9.0 (Graph Pad Software, Inc.) was used for the statistical analysis and graphing. Standard deviations and means were used to describe measurement data that followed a normal distribution. Baseline data analysis of mean ± standard deviation or median and quartile were used for quantitative data. The spacing was described, and the *t* test (or Wilcoxon test) was used to compare this between the groups. The paired *t* test was used for intra-group comparisons, the unpaired *t* test was used for comparisons between the groups, and the chi-squared test was used for gender comparisons between the groups. The Pearson correlation coefficient was used to analyze the correlation between the BBS, the TUGT, and the 10MWT in stroke-affected patients and the correlation between the BBS score and the peak plantar pressure and gait parameters of the bilateral limbs. Any *P* values < 0.05 were considered statistically significant.

## 3. Results

### 3.1. Comparison of peak plantar pressure and gait parameters in both groups

**FIGURE 1:**
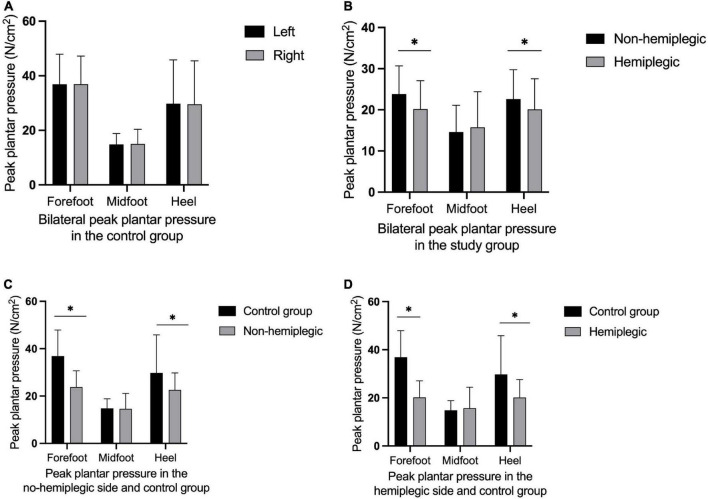
Peak plantar pressure in both group. There was no significant difference in bilateral peak plantar pressure in the control group **(A)** (*P* > 0.05). The peak plantar pressure of the forefoot and heel on the non-hemiplegic side were significantly higher than in the hemiplegic side in the study group **(B)** (*P* < 0.001). The peak plantar pressure of the forefoot and heel on the non-hemiplegic side of the study group was significantly lower than that in the control group **(C)** (*P* < 0.05). The peak plantar pressure of the forefoot and heel on the hemiplegic side of the study group was significantly lower than that in the control group **(D)** (*P* < 0.05). *Indicates significantly different.

**FIGURE 2:**
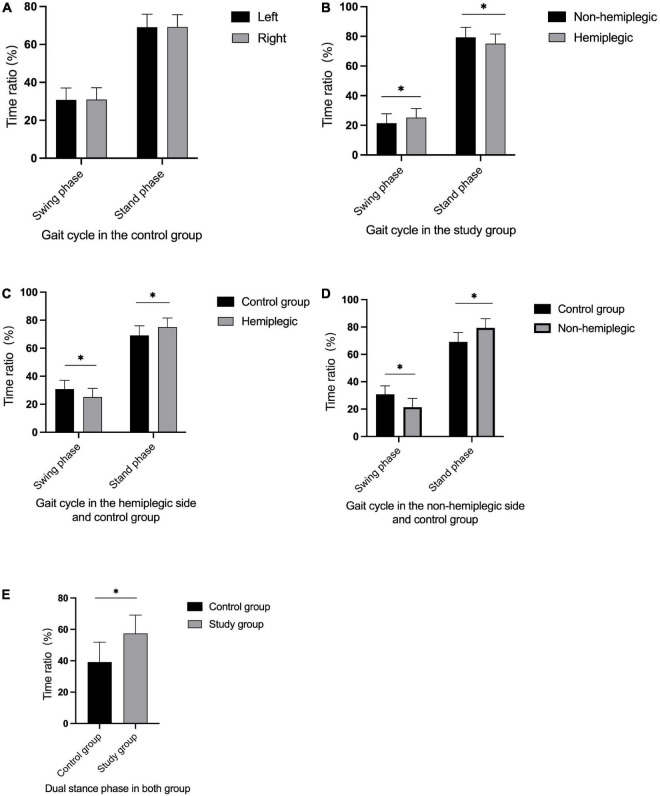
Gait cycle in both group. There was no significant difference in bilateral gait cycle in the control group **(A)** (*P* > 0.05). The non-hemiplegic swing and stand phase were significantly higher than in the hemiplegic side in the study group **(B)** (*P* < 0.001). The swing phase in the hemiplegic side in the study group was significantly lower than in the control group, while the stand phase in the hemiplegic side was higher than in the control group **(C)** (*P* < 0.05). The swing phase in the non-hemiplegic side in the study group was significantly lower than in the control group, while the stand phase in the non-hemiplegic side was higher than in the control group **(D)** (*P* < 0.05). The dual stand phase in the study group was higher than in the control group **(E)** (*P* < 0.001). *Indicates significantly different.

**FIGURE 3:**
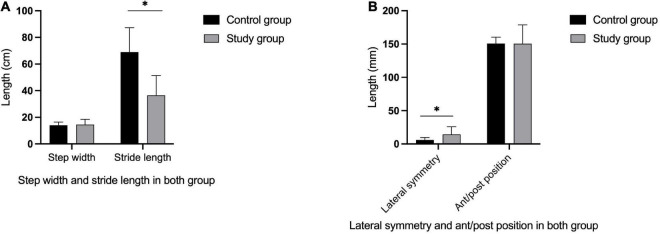
Gait parameters in both group. The stride length in the study group was significantly lower than in the control group **(A)** (*P* < 0.05), while there was no significant differences in step width between two groups **(A)** (*P* > 0.05). The lateral symmetry in the study group were higher than in the control group **(B)** (*P* < 0.05), while there was no significant differences in ant/post position between the two groups **(B)** (*P* > 0.05). *Indicates significantly different.

**FIGURE 4:**
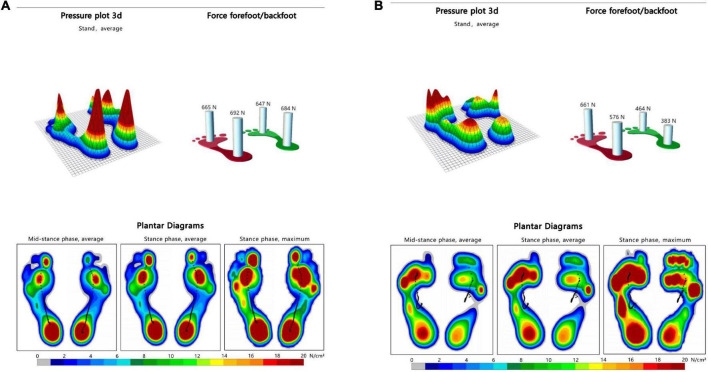
Dynamic plantar pressure model diagrams in both group. When healthy people walking, the plantar pressure on both sides were evenly distributed, showing obvious symmetry **(A)**. While stroke patients walking, bilateral plantar pressure were unevenly distributed, showing significant asymmetry **(B)**. Right side is the hemiplegic side in panel **(B)**, and the peak plantar pressure of the forefoot and heel in the hemiplegic side were lower than those in the non-hemiplegic side.

**FIGURE 5:**
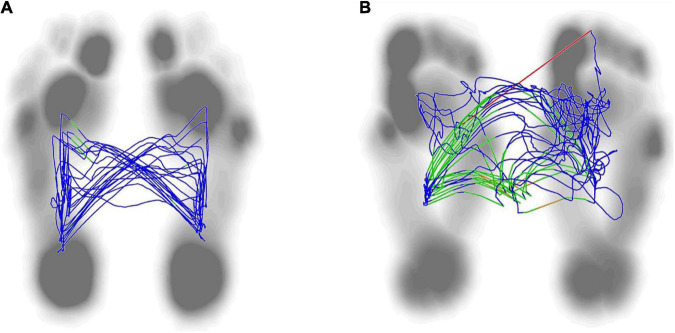
Lateral symmetry of center of plantar in both group. The normal center pressure trajectories distributed in a butterfly shape, with left-right symmetry **(A)**. The center pressure trajectory distribution of stroke patient was asymmetrical and shifting toward the hemiplegic side **(B)**.

**FIGURE 6:**
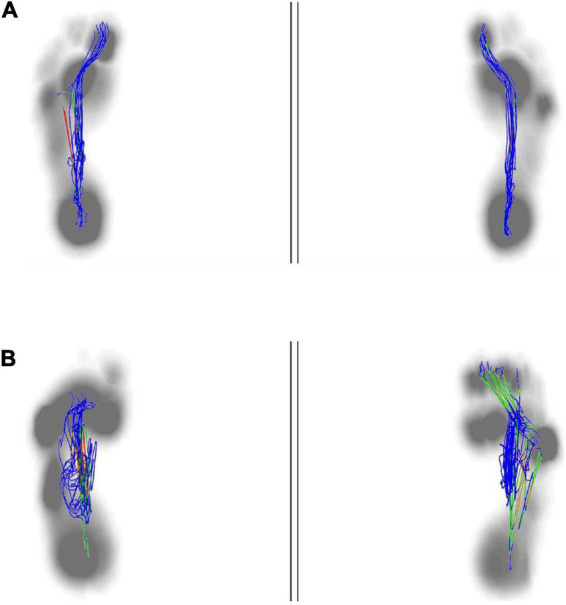
Anterior to posterior position of center of plantar. The anterior to posterior pressure line start from the heel to the big toe. The normal plantar pressure trajectory from anterior to posterior was almost overlapped **(A)**. The plantar pressure trajectory from anterior to posterior in stroke patient was not exactly overlapping, mainly concentrated in the midfoot, and was shorter than the healthy people **(B)**.

In the control group, there was no significant difference in bilateral peak plantar pressure and gait cycle (*P* > 0.05). There were no significant differences in step width and ant/post position between the two groups (*P* > 0.05). In addition, the peak plantar pressure of the forefoot and heel, stride length, swing phase in the study group were significantly lower than those in the control group (*P* < 0.05), while the stand phase, dual stand phase and the lateral symmetry in the study group were higher than those in the control group (*P* < 0.05) (Table 2 and Figures 1–3). The dynamic plantar pressure model diagram like Figure 4. The model diagram of COP just like Figures 5, 6.

**TABLE 2:** Comparison of peak plantar pressure and gait parameters in both groups.

Project	Control group		Study group	
	**Left**	**Right**	**Non-hemiplegic**	**Hemiplegic**
Forefoot (N/m^2^)	36.86 ± 11.07	36.93 ± 10.28	23.83 ± 6.85*	20.17 ± 6.91*^#^
Midfoot (N/m^2^)	14.80 ± 4.03	15.04 ± 5.33	14.6 ± 6.52	15.74 ± 8.68
Heel (N/m^2^)	29.75 ± 16.07	29.53 ± 15.94	22.62 ± 7.14*	20.11 ± 7.45*^#^
Swing phase (%)	30.79 ± 6.26	30.99 ± 6.17	21.42 ± 6.41*	25.2 ± 6.05*^#^
Stand phase (%)	69.12 ± 6.85	69.25 ± 6.46	79.36 ± 6.68*	75.13 ± 6.39*^#^
Dual stance phase (%)	39.22 ± 12.71		57.41 ± 11.66*	
Step width (cm)	14.03 ± 2.37		14.55 ± 3.94	
Stride length (cm)	68.94 ± 18.34		36.52 ± 14.84*	
Lateral symmetry (mm)	5.89 ± 3.72		14.49 ± 11.49*	
Anterior to posterior position (mm)	150.6 ± 9.52		150.6 ± 28.15	

Compared with the the control group, *indicates *P* < 0.05. Compared with the non-hemiplegic limb, ^#^indicates *P* < 0.05.

### 3.2. Comparison of peak plantar pressure and gait parameters between the non-hemiplegic side and the hemiplegic side in the study group

There was no significant difference in the peak plantar pressure of the midfoot between the non-hemiplegic side and the hemiplegic side (*P* > 0.05). However, the peak plantar pressure of the forefoot and heel on the non-hemiplegic side and the non-hemiplegic swing and stand phase were significantly higher than in the hemiplegic side in the study group (*P* < 0.05) (Table 2 and Figures 1–3).

### 3.3. Correlation analysis between the Berg Balance Scale score and peak plantar pressure and gait parameters in the study group

**FIGURE 7:**
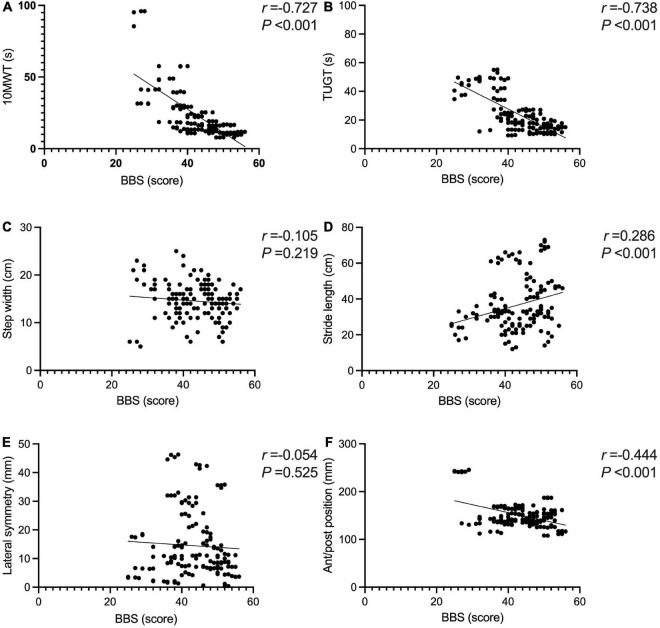
Correlations between the Berg Balance Scale score and gait parameters in the study group. The BBS and the 10MWT had a negative correlation **(A)** (*r* = −0.727, *P* < 0.001). The BBS and the TUGT had a negative correlation **(B)** (*r* = −0.738, *P* < 0.001). The BBS score was not correlated with step width **(C)** (*P* > 0.05). The BBS score was positively correlated with the stride length **(D)** (*r* = 0.286, *P* < 0.001). The BBS score was not correlated with lateral symmetry **(E)** (*P* > 0.05). The BBS score was negatively correlated with the ant/post position **(F)** (*r* = −0.444, *P* < 0.001). *Indicates significantly different.

**FIGURE 8:**
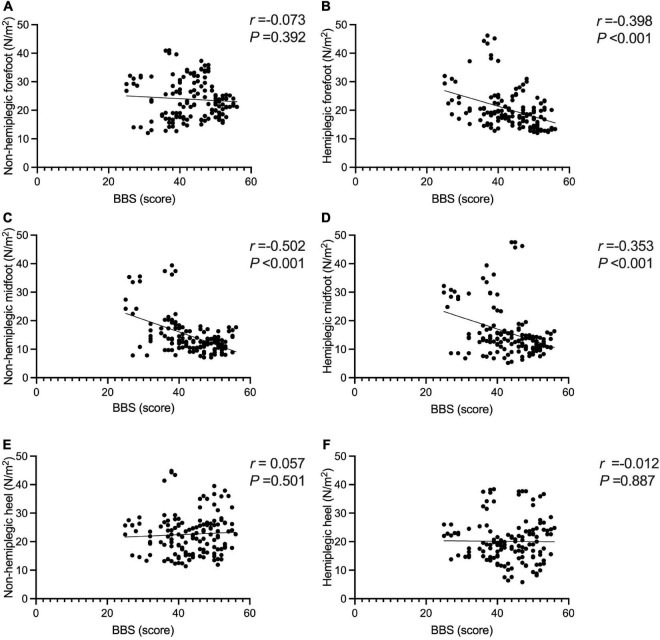
Correlations between the Berg Balance Scale score and peak plantar pressure in the study group. The BBS score was not correlated with the peak plantar pressure of the non-hemiplegic forefoot **(A)** (*P* > 0.05), but it was negatively correlated with the peak plantar pressure of the hemiplegic forefoot **(B)** (*r* =−0.398, *P* < 0.001). The BBS score was negatively correlated with the peak plantar pressure of the non-hemiplegic midfoot **(C)** (*r* =−0.502, *P* < 0.001) and the hemiplegic midfoot **(D)** (*r* =−0.353, *P* < 0.001). The BBS score was not correlated with the peak plantar pressure of the non-hemiplegic heel **(E)** (*P* > 0.05) and hemiplegic heel **(F)** (*P* > 0.05).

**FIGURE 9:**
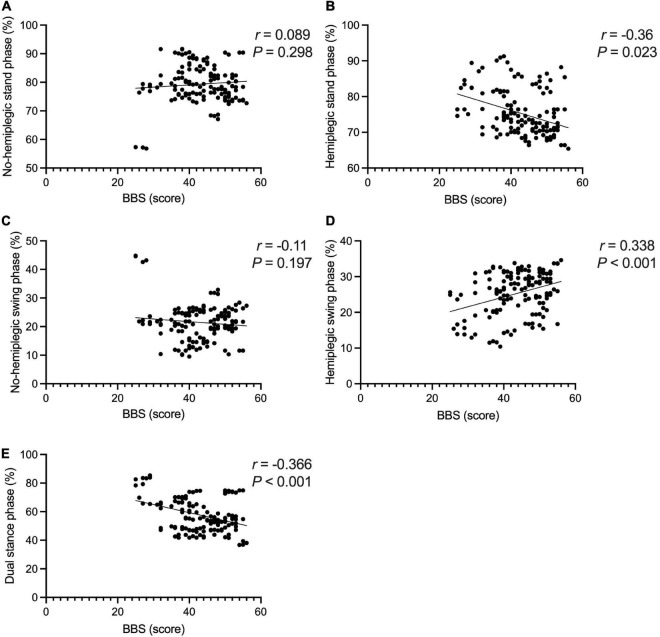
Correlations between the Berg Balance Scale score and gait cycle in the study group. The BBS score was not correlated with the non-hemiplegic stand phase **(A)** (*P* > 0.05), non-hemiplegic swing phase **(B)** (*P* > 0.05). The BBS score was negatively correlated with the hemiplegic stand phase **(C)** (*r* = −0.36, *P* = 0.023), hemiplegic swing phase **(D)** (*r* = −0.338, *P* < 0.001). The BBS score was negatively correlated with dual stand phase **(E)** (*r* = −0.366, *P* < 0.001).

The BBS and the 10MWT had a negative correlation (*r* = −0.727, *P* < 0.001), as did the BBS and the TUGT (*r* = −0.738, *P* < 0.001). The BBS score was not correlated with step width, or lateral symmetry (*P* > 0.05), but it was negatively correlated with the ant/post position (*r* = −0.444, *P* < 0.001) and positively correlated with the stride length (*r* = 0.286, *P* < 0.001). The BBS score was not correlated with the peak plantar pressure of the non-hemiplegic forefoot, non-hemiplegic heel, or hemiplegic heel (*P* > 0.05), but it was negatively correlated with the peak plantar pressure of the hemiplegic forefoot (*r* = −0.398, *P* < 0.001), hemiplegic midfoot (*r* = −0.353, *P* < 0.001), and the non-hemiplegic midfoot (*r* = −0.502, *P* < 0.001). The BBS score was not correlated with the non-hemiplegic stand phase, non-hemiplegic swing phase (*P* > 0.05), but it was negatively correlated with the hemiplegic stand phase (*r* = −0.36, *P* = 0.023), hemiplegic swing phase (*r* = 0.338, *P* < 0.001) and the dual stand phase (*r* = −0.366, *P* < 0.001) (Table 3 and Figures 7–9).

**TABLE 3:** Correlations between the Berg Balance Scale score and peak plantar pressure and gait parameters in the study group.

Project	Berg Balance Scale score	
	***r***	***P***
10-m walk test	-0.727	<0.001
Timed up-and-go test	-0.738	<0.001
Peak plantar pressure of hemiplegic forefoot	-0.398	<0.001
Peak plantar pressure of hemiplegic midfoot	-0.353	<0.001
Peak plantar pressure of hemiplegic heel	-0.012	0.887
Peak plantar pressure of non-hemiplegic forefoot	-0.073	0.392
Peak plantar pressure of non-hemiplegic midfoot	-0.502	<0.001
Peak plantar pressure of non-hemiplegic heel	0.057	0.501
Step width	-0.105	0.219
Stride length	0.286	0.0006
Lateral symmetry	-0.054	0.525
Anterior to posterior position	-0.444	<0.001
Hemiplegic stand phase	-0.36	0.023
Hemiplegic swing phase	0.338	<0.001
Non-hemiplegic stand phase	0.089	0.298
Non-hemiplegic swing phase	-0.11	0.197
Dual stance phase	-0.366	<0.001

## 4. Discussion

The gait patterns caused by cerebral infarction in different regions are dissimilar (Bhatt et al., 2018). This study mainly observed the relation between balance function and gait after cerebral infarction in the basal ganglia region.

Stroke-affected patients have abnormal gait, weakened physical control, swaying trunk, asymmetrical weight-bearing of both lower limbs, and reduced ability to shift their center of gravity, resulting in unstable walking and an increased risk of falling (Saleh et al., 2019).

This study compared the plantar pressures in both groups. The plantar pressure of the forefoot and heel of the hemiplegic side of the study group was significantly lower than that of the non-hemiplegic side. The bilateral plantar pressure of the study group was significantly lower than that of the control group. The plantar pressure of the midfoot in both groups did not change significantly. These results indicate that the plantar pressure of patients with basal ganglia cerebral infarction was abnormal, and the non-hemiplegic side also showed abnormal plantar pressure distribution due to the influence of the hemiplegic side.

This trial also compared the gait cycles of the two groups. In the study group, the hemiplegic side swing phase was higher than that of the non-hemiplegic side, while the hemiplegic side stand phase was lower than that on the non-hemiplegic side. In the study group, the swing phase was significantly lower while the stand and dual stance phases were significantly higher than those in the control group, indicating that the basal ganglia region stroke-affected patients had abnormal gait and poor walking stability. To avoid falls, the time taken for the forefoot to leave the ground to the heel to land is shortened, resulting in prolonged unilateral support. This study showed that the stride length of the patients in the study group was significantly reduced compared with that of the control group, and the lateral symmetry was significantly increased. This indicates that stroke-affected patients have a short stride length and a gait with poor left–right symmetry, and therefore suggests a high risk of falling, even if they could walk independently.

In a meta-analysis, van Duijnhoven et al. (2016) reported that giving patients balance training and exercise therapy, such as the center-of-gravity metastasis, could improve their balance function and walking ability. The current study showed that the BBS score was negatively correlated with the 10MWT and the TUGT. This indicated that the patient’s balance function was related to their walking function, which follows previous studies. Chen et al. (2005) argued that patients with hemiplegia have weak limbs during the swing of the lower limbs on the hemiplegic side and need to provide compensation from the non-hemiplegic side, which reduces the speed; also, the 10MWT could reflect the dynamic changes in the pace of patients with hemiplegia during walking. The present study showed that better balance function of stroke-affected patients was associated with the shorter time and faster pace required to complete the 10MWT. The TUGT can assess the risk of falling during walking in stroke-affected patients and the ability of sit to stand transfer and posture control (Pérez-de la Cruz, 2021). The better the balance function of patients, the stronger the posture control ability, the higher the walking stability, and the lower the risk of falling.

The results revealed no significant correlation between the BBS score and step width, but revealed positive correlation between the BBS score and stride length in stroke-affected patients. Koch et al. conducted a gait analysis of stroke-affected patients, finding these patients had balance disorders and unstable walking. Still, they did not analyze the correlation between the balance function and stride length and step width (Koch et al., 2019). This may suggest that the gait characteristics of sensory and non-sensory disorder balance dysfunction are different. Hong et al. (2020) reported that the motor function of the hemiplegic lateral limbs in stroke-affected patients was impaired, and to maintain better gait stability during walking, the patient’s step width was significantly increased and the stride length was reduced. However, the effect of non-sensory balance function on stroke-affected patients was not ruled out. Lewek et al. (2014) argued that the BBS score was correlated with stride length, and walking step width could indicate the balance ability in stroke-affected patients.

After a stroke, the asymmetric gait during walking activates a compensation mode through muscle movement, posture, and gait training, which is conducive to the emergence of normal gait (Beyaert et al., 2015). Yang et al. (2018) reported that improving the gait symmetry of stroke-affected patients can advance their walking ability. Forghany reported that stroke-affected patients acquired a pattern of bipedal asymmetry when walking, which was related to limited walking. Hornby et al. (2019) believed that improving the walking ability of stroke-affected patients made it possible to improve gait symmetry, especially patients’ confidence in obtaining balance.

The present study suggested that the balance function of stroke-affected patients affects their gait symmetry. The balance function mainly affects the support line of the ant/post position. Better balance function of the patient may be associated with a stronger ability to support the front and back positions and better front–back symmetry, eventually resulting in a shorter length of the support line in the front and back positions. No significant correlation was found between equilibrium function and lateral symmetry, which needs to be explored further.

The present study analyzed the correlation between the BBS score and dynamic peaked plantar pressure. The results showed there was no obvious correlation between the BBS score and non-paralytic forefoot and heel, and hemiplegic midfoot and heel, but a negative correlation with hemiplegic forefoot and midfoot and non-paralytic midfoot was found. Rogers et al. (2020) suggested the changes in plantar pressure during walking in patients to improve walking ability should be explored. Forghany et al. (2015) reported that the plantar pressure distribution of stroke-affected patients is asymmetric. In the support phase, the lateral forefoot and heel are under greater pressure, while the hemiplegic heel is under the most pressure, and the midfoot and forefoot are under less pressure (Forghany et al., 2015). Lou et al. (2020) found that, after the patient’s walking ability improved, the peak plantar pressure in the hemiplegic forefoot increased and the peak plantar pressure on the non-hemiplegic heel was lower than before.

Few reports exist about the changes in balance function and plantar pressure during walking. This study further explored the changes in plantar pressure in patients with stroke balance function during walking. It is believed that the balance function after stroke is closely related to the hemiplegic forefoot and midfoot and non-paralyzed midfoot. When stroke-affected patients walk, due to hemiplegic lateral foot inversion and toe flexion (Park et al., 2021), the plantar pressure cannot be transferred to the inner side of the forefoot in the support phase, the pressure is concentrated on the outside of the forefoot, and the peak pressure on the forefoot is higher. Patients with a better balance function can relieve the foot-inverted mode. In the support phase, the contact area between the forefoot and the ground becomes larger, resulting in a decrease in the peak pressure of the forefoot. The higher the balance function, the lower the peak pressure of the forefoot in the support phase.

In the present study, there was no obvious correlation between the BBS score and the stance phase, swing phase of the non-hemiplegic limbs, while the BBS score was negatively correlated with the stance and swing phases of the hemiplegic limbs and dual stance phase. Wang et al. (2021) reported that as the pressure of hemiplegic forefoot in stroke-affected patients improved, the active dorsiflexion of the hemiplegic foot was promoted, the center of gravity was easier to move forward, the walking ability improved, and the balance ability improved. In et al. (2017) improved the patient’s knee extension ability, strengthened the contact area of the feet in the stance phase, and strengthened the dorsiflexion to reduce the time of the hemiplegic stance and swing phases, improve the patient’s balance function, and improve their walking ability. The present study’s findings are consistent with those of previous studies. The better the balance function, the more sufficient the center of gravity shift and the more adequate the ankle dorsiflexion during the foot landing (Persson et al., 2011).

The characteristics of gait under balance dysfunction caused by different reasons are dissimilar. The mature research is mostly about gait characteristics under proprioception impairment, and the effect of hemiplegic gait or balance function under abnormal postural control on gait is unclear (Luque-Moreno et al., 2019). The plantar pressure distribution during walking reflects the abnormal gait of patients with walking dysfunction, the uneven distribution of body weight, and the process of pressure changes in both limbs (Lund et al., 2018). Abnormal distribution of plantar pressure increases the risk of injury to the patient’s waist, knees, calves, ankles, and feet, further increasing the risk of falls during walking training and affecting the recovery of the patient’s walking ability (Yang et al., 2014).

Existing studies have shown that balance function affects walking in stroke-affected patients (An and Shaughnessy, 2011), and the plantar pressure distribution reflects the patient’s ability to walk (Kimura et al., 2022). However, there are few studies exploring balance function and plantar pressure, and the distribution of balance ability and plantar pressure during walking in stroke-affected patients is unclear (Bower et al., 2019). When stroke-affected patients with balance dysfunction perform walking training, their balance function affects their posture control, transfer ability, and the length of the limb support and double support phases of the hemiplegic side during walking, increasing the asymmetry of their hemiplegic gait. This affects their gait pace and stability and increases the risk of falling. Walking training should pay attention to the plantar pressure distribution of the forefoot and midfoot on the hemiplegic side and improve the symmetry of the ant/post positions to progress the patient’s walking ability. This provides a new direction for subsequent rehabilitation treatment.

This study has certain limitations: (1) the small sample size may mean the changes in different variables may have been too small to be significant, impacting the final result and (2) there is a difference between the running platform and a normal walking mode, and the measurement of the plantar pressure may have caused errors and affected the test results. In future research, the sample size should be increased, the authority of the research should be enhanced, and the patient’s walking ability should be improved through the patient’s plantar pressure and front–back position symmetry to provide a new direction to treat stroke-affected patients.

## 5. Conclusion

After cerebral infarction in the basal ganglia, there is typically asymmetry in the pressure of the forefoot and the ant/post position, which significantly affects the balance function. It is necessary to pay attention to the distribution of hemiplegic forefoot plantar pressure, the control of gait phase, and the processing of the ant/post position to better improve balance and posture control, reducing the risk of falling.

## Data availability statement

The original contributions presented in this study are included in the article/supplementary material, further inquiries can be directed to the corresponding author.

## Ethics statement

The study was approved by the Ethics Committee of Beijing Tiantan Hospital Affiliated with Capital Medical University (Approval No. KY2021-040-02). The patients/participants provided their written informed consent to participate in this study.

## Author contributions

SL: methodology, validation, formal analysis, and writing—original draft preparation. HY: conceptualization, writing—review and editing, supervision, and project administration. ZW: software and data curation. PD: resources and visualization. All authors contributed to manuscript revision, read, and approved the submitted version.
